# An efficient approach to estimate the risk of coronary artery disease for people living with HIV using machine-learning-based retinal image analysis

**DOI:** 10.1371/journal.pone.0281701

**Published:** 2023-02-24

**Authors:** Grace Lui, Ho Sang Leung, Jack Lee, Chun Kwok Wong, Xinxin Li, Mary Ho, Vivian Wong, Timothy Li, Tracy Ho, Yin Yan Chan, Shui Shan Lee, Alex PW Lee, Ka Tak Wong, Benny Zee

**Affiliations:** 1 Department of Medicine and Therapeutics, Prince of Wales Hospital, The Chinese University of Hong Kong, Shatin, Hong Kong SAR; 2 Stanley Ho Centre for Emerging Infectious Diseases, The Chinese University of Hong Kong, Shatin, Hong Kong SAR; 3 Department of Imaging and Interventional Radiology, Prince of Wales Hospital, Shatin, Hong Kong SAR; 4 Centre for Clinical Research and Biostatistics, The Jockey Club School of Public Health and Primary Care, The Chinese University of Hong Kong, Shatin, Hong Kong SAR; 5 Department of Chemical Pathology, The Chinese University of Hong Kong, Shatin, Hong Kong SAR; 6 Department of Ophthalmology, Prince of Wales Hospital, Shatin, Hong Kong SAR; 7 Laboratory of Cardiac Imaging and 3D Printing, Li Ka Shing Institute of Health Science, The Chinese University of Hong Kong, Shatin, Hong Kong SAR; Kurume University School of Medicine, JAPAN

## Abstract

**Background:**

People living with HIV (PLWH) have increased risks of non-communicable diseases, especially cardiovascular diseases. Current HIV clinical management guidelines recommend regular cardiovascular risk screening, but the risk equation models are not specific for PLWH. Better tools are needed to assess cardiovascular risk among PLWH accurately.

**Methods:**

We performed a prospective study to determine the performance of automatic retinal image analysis in assessing coronary artery disease (CAD) in PLWH. We enrolled PLWH with ≥1 cardiovascular risk factor. All participants had computerized tomography (CT) coronary angiogram and digital fundus photographs. The primary outcome was coronary atherosclerosis; secondary outcomes included obstructive CAD. In addition, we compared the performances of three models (traditional cardiovascular risk factors alone; retinal characteristics alone; and both traditional and retinal characteristics) by comparing the area under the curve (AUC) of receiver operating characteristic curves.

**Results:**

Among the 115 participants included in the analyses, with a mean age of 54 years, 89% were male, 95% had undetectable HIV RNA, 45% had hypertension, 40% had diabetes, 45% had dyslipidemia, and 55% had obesity, 71 (61.7%) had coronary atherosclerosis, and 23 (20.0%) had obstructive CAD. The machine-learning models, including retinal characteristics with and without traditional cardiovascular risk factors, had AUC of 0.987 and 0.979, respectively and had significantly better performance than the model including traditional cardiovascular risk factors alone (AUC 0.746) in assessing coronary artery disease atherosclerosis. The sensitivity and specificity for risk of coronary atherosclerosis in the combined model were 93.0% and 93.2%, respectively. For the assessment of obstructive CAD, models using retinal characteristics alone (AUC 0.986) or in combination with traditional risk factors (AUC 0.991) performed significantly better than traditional risk factors alone (AUC 0.777). The sensitivity and specificity for risk of obstructive CAD in the combined model were 95.7% and 97.8%, respectively.

**Conclusion:**

In this cohort of Asian PLWH at risk of cardiovascular diseases, retinal characteristics, either alone or combined with traditional risk factors, had superior performance in assessing coronary atherosclerosis and obstructive CAD.

**Summary:**

People living with HIV in an Asian cohort with risk factors for cardiovascular disease had a high prevalence of coronary artery disease (CAD). A machine-learning-based retinal image analysis could increase the accuracy in assessing the risk of coronary atherosclerosis and obstructive CAD.

## Background

Effective and durable anti-retroviral therapy allows us to witness tremendous improvement in life expectancy in people living with HIV (PLWH). However, substantial morbidity and mortality in PLWH are due to non-communicable diseases, such as cardiovascular diseases.

PLWH had a two-fold increased risk of cardiovascular diseases [1]. The global burden of cardiovascular diseases attributable to HIV has tripled since the 1990s, with sub-Saharan Africa and Asia-Pacific regions being the most affected areas [1]. Despite significant improvements in the management of HIV and its comorbidities over the last two decades. Recent population-based studies consistently showed that PLWH had higher cardiovascular and cerebrovascular disease risks [2, 3]. PLWH also carries a higher prevalence of risk factors for cardiovascular diseases, such as hypertension and diabetes [2]. Studies performed in Asia also showed that PLWH had high risks of coronary artery disease (CAD), partly attributable to the high prevalence of cardiovascular disease risk factors, suboptimal screening, and suboptimal management of these risk factors, especially in general low- and middle-income settings [4].

Current HIV clinical management guidelines recommend regular cardiovascular risk screening in PLWH; however, the best risk prediction model for PLWH is uncertain [5, 6]. Although various cardiovascular disease risk prediction functions are currently available. These algorithms have primarily been developed in non-HIV-infected populations. Therefore, they could not accurately predict risk in PLWH, possibly due to differences in pathogenesis underlying cardiovascular disease [7]. Moreover, many of these functions, including HIV-specific prediction models, have not been adequately validated in Asian populations of PLWH [4].

Retinal vascular characteristics have been recognized to encompass features associated with systemic diseases, such as diabetes and hypertension. Recent research has demonstrated a broader breadth of the application of retinal biomarkers for diagnostic, monitoring, and prognostic purposes in a wide range of chronic diseases [8]. Recently, retinal image characteristics have been shown to be closely linked with multiple cardiovascular risk factors and major cardiovascular events [9]. In particular, several retinal vascular characteristics, including arteriolar and venular calibre, curvature tortuosity, and branching complexity, were shown to have associations with CAD [10, 11].

Traditionally, manual interpretation of retinal images was heavily operator-dependent and time-consuming and was subjected to measurement error. Recently, the availability of automated models has shown superior accuracy and has transformed the practicality of adopting the assessment of retinal characteristics into routine clinical use [8, 12, 13]. However, most studies often involved a limited number of vascular characteristics [14]. In contrast, contemporary computing methods allow efficient and accurate measurements of a broad spectrum of retinal microvasculature characteristics. The retinal characteristics include vascular calibre, tortuosity, density, and branching complexity [15]. Retinal image analysis also has the advantages of being a simple, non-invasive procedure, requiring minimal operator training.

The application of retinal image analysis in assessing the risk of cardiovascular diseases has not yet been evaluated in PLWH. Also, HIV infection *per se* and associated opportunistic diseases cause various retinal abnormalities [16]. Therefore, studies showing a correlation between retinal vascular characteristics and cardiovascular diseases performed in non-HIV-infected populations might not be generalizable to PLWH. This study aimed to determine the prevalence of CAD among high-risk PLWH in a predominantly Asian population and determine the performance of machine-learning-based retinal image analysis in assessing the risk of CAD in PLWH compared to traditional risk prediction tools.

## Methods

We performed a prospective study on CAD in an Asian population of PLWH with atherosclerotic risk factors at the Prince of Wales Hospital Infectious Diseases clinic in Hong Kong from February 2019 to February 2021. The primary outcome is coronary atherosclerosis; secondary outcomes include the presence of any coronary artery calcium (CAC), significant CAC, and obstructive CAD.

We enrolled PLWH aged 30 years or above with either chest pain or the presence of one or more risk factors for cardiovascular disease. Cardiovascular disease risk factors included hypertension, diabetes mellitus, dyslipidemia (defined by total cholesterol ≥6.2 mmol/L, HDL cholesterol ≤0.9 mmol/L, triglyceride ≥2.3 mmol/L, or use of the lipid-lowering drug) [17], current smoker, obesity (defined by body mass index ≥27 kg/m^2^) [18], and family history of CAD (defined by a first-degree relative with myocardial infarction before age 50 years) [17]. Patients with previously diagnosed CAD, creatinine clearance < 60mL/min, allergy to intravenous contrast, and pregnancy were excluded. All participants provided written informed consent. The study was approved by the Joint Chinese University of Hong Kong-New Territories East Cluster Clinical Research Ethics Committee.

We collected demographic and clinical information, including HIV-related clinical data and comorbidities. We measured body weight, height, waist circumference, and blood pressure. After at least 8 hours of fasting, we collected blood samples for glucose, HbA1c, insulin, cholesterol, triglyceride, creatinine, C reactive protein, fibrinogen, D-dimer, sCD14, sCD163, and adiponectin.

We calculated cardiovascular risk using four cardiovascular risk prediction functions for each participant. They included Framingham risk score for 10-year coronary heart disease risk [19], QRISK3 [20], Pooled Cohort ASCVD risk equations [21], and 10-year cardiovascular disease risk using the Data-collection on Adverse Effects of Anti-HIV Drugs (D:A:D) study (DAD) cohort risk prediction [22]. A prediction of <10% was considered a low risk of cardiovascular disease.

All participants underwent a coronary CT angiogram, which included a non-contrast CT for calcium scoring and subsequently contrast-enhanced CT with cardiac gating for coronary angiography. We obtained a CAC score from the non-contrast CT and quantified it using the Agatston method [23]. The presence of any CAC was defined as Agatston score > 0, and significant CAC was defined as Agatston score ≥ 100. We assessed the coronary plaque burden, including the presence, the site, composition (calcified, non-calcified, or mixed), and degree of stenosis of the plaques following the Society of Cardiovascular Computed Tomography guidelines [24]. In brief, stenosis was categorized as normal or minimal (0–25%), mild (26% - 50%), moderate (51% - 75%), severe non-subtotal occlusion (76% - 90%), and severe subtotal occlusion (91–99%). Coronary atherosclerosis was defined as the presence of any plaques in one or more coronary artery segments, while moderate to severe stenosis of the lumen was considered as obstructive CAD.

A trained research nurse acquired digital fundus photographs using a Canon CR2-AF non-mydriatic retinal camera from both eyes of each participant. We then assessed retinal characteristics, for example, retinal vessel measurements, arteriole-venous nicking, arteriole occlusion, haemorrhages, exudates, tortuosity, bifurcation coefficients, asymmetry of branches, and bifurcation angles. The definitions of the retinal parameters were previously presented in detail [11, 25].

### Statistical methods

We performed these measurements using R (University of Auckland, Auckland) and Matlab (MathWorks, Massachusetts, USA) computer software. The methods included fractal analysis, high-order spectra analysis, and statistical texture analysis [25].

We presented descriptive statistics for the baseline characteristics. We compared demographic, clinical, and retinal characteristics between those with and without primary and secondary outcomes using an independent two-sample Student t-test and Mann Whitney U test for continuous variables and a chi-square test for categorical variables. Using stepwise logistic regression analyses, we determined the associations of traditional cardiovascular risk factors and retinal characteristics with the primary and secondary outcomes. Three different models were evaluated: Model 1 included clinical characteristics regarded as traditional cardiovascular risk factors in PLWH; model 2 included retinal characteristics only; model 3 included both the traditional cardiovascular risk factors and retinal characteristics. All covariates with a p-value less than 0.1 were kept in the final model. We compared the performances of different models by comparing the area under the curve (AUC) of receiver operating characteristic (ROC) curves using the Delong method [26] The sensitivity and specificity of the models will also be calculated.

**Fig 1:**
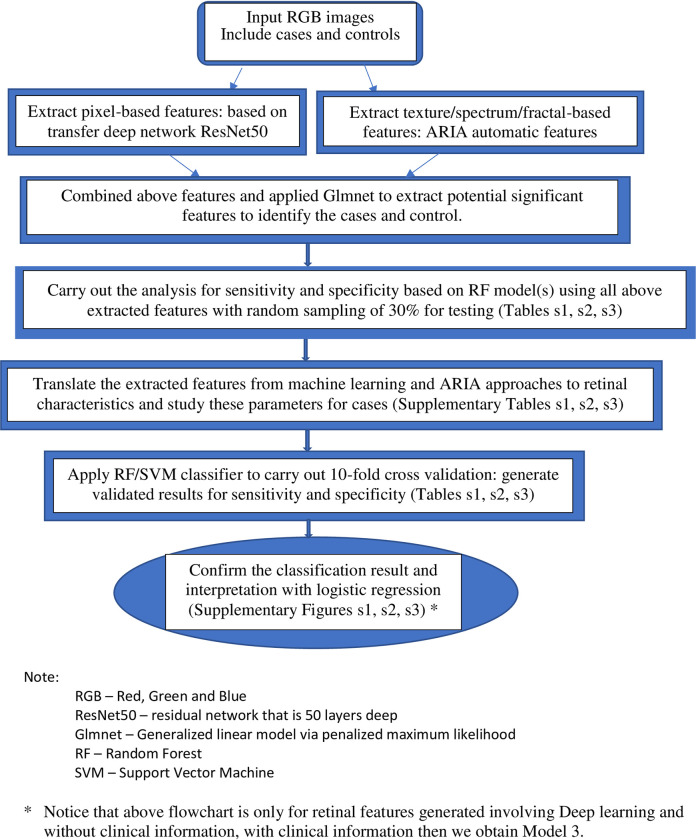
Flowchart of the method for the development of the classification models.

For the classification analysis, we used machine learning and deep learning techniques. Using Matlab, we first applied transfer deep network ResNet50 convolutional neural network with retinal images as input, and the outputs were features generated at the layer of ‘’fc1000_softmax’’, based on pixels associated with the specific outcome status [27]. We also extracted the texture/spectrum/fractal-based features that are associated with the specific outcome by using the automatic retinal image analysis (ARIA) algorithm written in Matlab [28]. We then used the Glmnet approach to select significant features based on the penalised maximum likelihood by using R and Matlab [29, 30]. These refined features are highly associated with the specific outcome. Finally, we translated the features extracted from the aforementioned machine learning approaches to commonly used retinal characteristics measured from the images using ImageJ. This part of the analysis helped enhance our understanding of retinal characteristics that contribute to the classification and identification of the specific outcome and was performed with SPSS. For the validation, we applied a 10-fold cross-validation method by using a support vector machine (SVM) algorithm for testing datasets that were not used in the training of the model [30, 31]. This was performed by partitioning the dataset and using a subset to train the algorithm, and the remaining subset of data for testing. Each time we ran the cross-validation analysis, we used 10% of the data for testing that were not used at all in the training data. The advantage of this method is that the data used for testing in each run were excluded from the specific training models for the purpose of validation to reduce the problem of overfitting and overestimation of the sensitivity and specificity. Because cross-validation does not use all of the data to build a model, it is a commonly used method to prevent overfitting during training. **Fig 1** shows the flowchart of the described methodology.

## Results

This study enrolled 120 participants during the recruitment period. Five participants were excluded from the analyses. Among them, 3 participants were excluded due to the unavailability of good quality retinal images, 1 participant was deceased, and 1 participant was lost to follow-up after recruitment without completing all study procedures. Among the 115 participants included in the analyses, the mean (±standard deviation) age was 54±10 years, 89% were male, the median (interquartile range/IQR) duration of HIV diagnosis was 12 (7–17) years, 95% had undetectable serum HIV RNA, and the median (IQR) CD4 count was 632 (451–840) cells/mm^3^. Among this cohort, 45% had hypertension, 40% had diabetes, 45% had dyslipidemia, and 55% had obesity. In addition, chest pain and dyspnea were present in 17% and 18% of participants. Their detailed demographic and clinical characteristics are shown in **Tables 1 and 2**.

**Table 1:** Demographic and clinical characteristics in participants with and without coronary atherosclerosis^1^.

Characteristics	All participants (N = 115)	No coronary atherosclerosis (N = 44)	Coronary atherosclerosis (N = 71)	P-value
Age (years)	53.7±9.5	49.9±9.7	56.1±8.7	0.001
Male	102 (88.7%)	35 (79.6%)	67 (94.4%)	0.030
Current smoker	27 (23.7%)	14(31.82)	13(18.31)	0.105
Family history of premature myocardial infarction	3 (2.6%)	2(4.55)	1(1.41)	0.557
Diabetes mellitus	46 (40.0%)	16 (36.4%)	30(42.3%)	0.530
Hypertension	52 (45.2%)	15 (34.1%)	37 (52.1%)	0.059
Dyslipidemia	52 (45.2%)	12 (27.3%)	40 (56.3%)	0.002
Obesity	63 (54.8%)	24 (54.6%)	39 (54.9%)	0.968
Hepatitis B	9 (7.8%)	5 (11.4%)	4 (5.6%)	0.450
Hepatitis C	3 (2.6%)	2 (4.6%)	1 (1.4%)	0.557
Chest pain	20 (17.4%)	6 (13.6%)	14 (19.7%)	0.403
Dyspnoea	21 (18.3%)	8 (18.2%)	13 (18.3%)	0.986
Duration of HIV diagnosis (years)	12 (7–17)	12 (8–5)	13 (7–17)	0.928
History of AIDS	24 (20.9%)	11 (25.0%)	13 (18.3%)	0.391
Duration of antiretroviral therapy (years)	9 (5–13)	9 (6–13)	9 (5–13)	0.714
Current anti-retroviral drugs				
Tenofovir	78 (67.8%)	29 (65.9%)	49 (69.0%)	0.729
Abacavir	31 (27.0%)	13 (29.5%)	18 (25.4%)	0.622
NNRTI	27 (23.5%)	10 (22.7%)	17 (23.9%)	0.881
Protease inhibitor	19 (16.5%)	6 (13.6%)	13 (18.3%)	0.512
Integrase strand transfer inhibitor	74 (64.3%)	29 (65.9%)	45 (63.4%)	0.783
Body weight (kg)	74.0±13.9	75.4±15.1	73.1±13.2	0.381
Body mass index (kg/m^2^)	26.5±7.2	27.9±10.3	25.6±4.3	0.366
Waist circumference (cm)	91.6±11.6	91.9±12.8	91.4±10.8	0.805
Systolic blood pressure (mmHg)	133±18	130±16	136±19	0.247
Diastolic blood pressure (mmHg)	86±11	86±12	87±11	0.67
CD4 count (cells/mm^3^)	632 (451–840)	666 (496–840)	615 (419–865)	0.295
CD4:CD8 ratio	0.83±0.36	0.95±0.36	0.76±0.35	0.003
HIV RNA <50 copies/mL	109 (94.8%)	42(95.45)	67(94.37)	1.000
Total cholesterol (mmol/L)	4.70±1.08	4.77±1.03	4.65±1.12	0.559
HDL (mmol/L)	1.19±0.38	1.25±0.37	1.15±0.39	0.063
LDL (mmol/L)	2.55±0.89	2.71±0.87	2.44±0.89	0.120
Triglycerides (mmol/L)	1.9 (1.4–2.8)	1.8 (1.2–2.4)	2.2 (1.5–3.0)	0.057
Glucose (mmol/L)	5.9 (5.2–6.8)	5.6 (5.1–6.5)	6.1 (5.3–7.1)	0.124
HbA1C (mmol/L)	5.9 (5.6–6.7)	5.9 (5.6–6.5)	6.0 (5.6–6.7)	0.274
HOMA-IR	2.3 (1.2–4.4)	2.2 (1.1–3.1)	2.3 (1.3–4.8)	0.156
Creatinine (μmol/L)	88.2±16.5	85.6±16.2	89.7±16.6	0.195
D-dimer (ng/mL)	259 (177–353)	224 (164–328)	263 (177–351)	0.405
Fibrinogen (g/L)	2.99±0.58	3.02±0.56	2.96±0.59	0.591
C reactive protein (mg/L)	1.4 (0.6–3.0)	1.7 (0.6–3.6)	1.4 (0.6–2.3)	0.224
sCD163 (ng/mL)	626±144	622±150	629±141	0.943
sCD14 (pg/mL)	2228 (2131–2371)	2232 (2077–2402)	2226 (2139–2344)	0.820
Adiponectin (ng/mL)	1305 (674–3277)	1591 (744–3529)	1233 (587–3115)	0.299

^1^Data are presented as number (percentage), mean ± standard deviation, or median (interquartile range), as appropriate

**Table 2:** Demographic and clinical characteristics in participants with and without obstructive coronary artery disease (CAD)^#^.

Characteristics	All participants (N = 115)	No obstructive CAD (N = 92)	Obstructive CAD (N = 23)	P-value
Age (years)	53.7±9.5	52.09±9.13	60.22±8.40	<0.001
Male	102 (88.7%)	81(88.04)	21(91.3)	0.941
Current smoker	27 (23.7%)	23 (25.3%)	4 (17.4%)	0.427
Family history of premature myocardial infarction	3 (2.6%)	2 (2.2%)	1 (4.4%)	0.491
Diabetes mellitus	46 (40.0%)	36 (39.1%)	10 (43.5%)	0.703
Hypertension	52 (45.2%)	39 (42.4%)	13 (56.5%)	0.223
Dyslipidemia	52 (45.2%)	39 (42.4%)	13 (56.5%)	0.223
Obesity	63 (54.8%)	53 (57.6%)	10 (43.5%)	0.223
Hepatitis B	9 (7.8%)	9 (9.8%)	0 (0%)	0.259
Hepatitis C	3 (2.6%)	3 (3.3%)	0 (0%)	1.000
Chest pain	20 (17.4%)	15 (16.3%)	5 (21.7%)	0.758
Dyspnoea	21 (18.3%)	12 (13.0%)	9 (39.1%)	0.010
Duration of HIV diagnosis (years)	12 (7–17)	12 (7–16)	13(6–19)	0.661
History of AIDS	24 (20.9%)	20 (21.7%)	4 (17.4%)	0.863
Duration of antiretroviral therapy (years)	9 (5–13)	9 (6–13)	9 (3–14)	0.875
Current anti-retroviral drugs				
Tenofovir	78 (67.8%)	61 (66.3%)	17 (73.9%)	0.485
Abacavir	31 (27.0%)	27 (29.3%)	4 (17.4%)	0.248
NNRTI	27 (23.5%)	23 (25.0%)	4 (17.4%)	0.441
Protease inhibitor	19 (16.5%)	15 (16.3%)	4 (17.4%)	1.000
Integrase strand transfer inhibitor	74 (64.3%)	59 (64.1%)	15 (62.5%)	0.922
Body weight (kg)	74.0±13.9	75.1±13.9	69.3±13.4	0.119
Body mass index (kg/m^2^)	26.5±7.2	26.9±7.7	24.8±4.4	0.231
Waist circumference (cm)	91.6±11.6	91.9±11.4	90.4±12.4	0.605
Systolic blood pressure (mmHg)	133±18	133±16	136±23	0.534
Diastolic blood pressure (mmHg)	86±11	86±12	86±9	0.952
CD4 count (cells/mm^3^)	632 (451–840)	623(455–839.5)	686(427–865)	0.729
CD4:CD8 ratio	0.83±0.36	0.85±0.38	0.78±0.3	0.418
HIV RNA <50 copies/mL	109 (94.8%)	87 (94.6%)	22 (95.7%)	1.000
Total cholesterol (mmol/L)	4.70±1.08	4.81±1.04	4.25±1.14	0.027
HDL (mmol/L)	1.19±0.38	1.21±0.4	1.12±0.32	0.317
LDL (mmol/L)	2.55±0.89	2.64±0.86	2.16±0.93	0.021
Triglycerides (mmol/L)	1.9 (1.4–2.8)	1.9 (1.4–2.9)	2.2 (1.1–2.8)	0.997
Glucose (mmol/L)	5.9 (5.2–6.8)	5.9 (5.2–6.9)	5.9 (5.4–6.3)	0.761
HbA1C (mmol/L)	5.9 (5.6–6.7)	5.9 (5.6–6.7)	5.9 (5.7–6.6)	0.718
HOMA-IR	2.3 (1.2–4.4)	2.2 (1.2–3.9)	2.9 (1.1–5.3)	0.246
Creatinine (μmol/L)	88.2±16.5	88.61±16.94	86.3±14.65	0.551
D-dimer (ng/mL)	259 (177–353)	240.0(164.0–333.5)	267.5 (190.0–448.0)	0.146
Fibrinogen (g/L)	2.99±0.58	2.99±0.54	2.98±0.71	0.965
C reactive protein (mg/L)	1.4 (0.6–3.0)	1.4(0.6–3.0)	1.4(0.6–3.1)	0.960
sCD163 (ng/mL)	626±144	611±146	686±119	0.024
sCD14 (pg/mL)	2228 (2131–2371)	2232 (2127–2392)	2212 (2139–2321)	0.756
Adiponectin (ng/mL)	1305 (674–3277)	1378 (696–3444)	1094 (369–2799)	0.277

^#^Data are presented as number (percentage), mean ± standard deviation, or median (interquartile range), as appropriate

Seventy-one participants (61.7%) had coronary atherosclerosis; also, these 71 participants were found to have a presence of any CAC. Thirty-five participants (30.4%) had significant CAC, and 23 (20.0%) had obstructive CAD. Coronary atherosclerosis was associated with male gender, older age, dyslipidemia, hypertension, lower CD4:CD8 ratio, lower HDL cholesterol, and higher triglyceride (**Table 1**). Obstructive CAD was associated with older age, dyspnea, lower total cholesterol, lower LDL cholesterol, and higher sCD163 (**Table 2**). Significant CAC was associated with older age, dyslipidemia, and lower LDL cholesterol (**S1 Table**).

**Fig 2:**
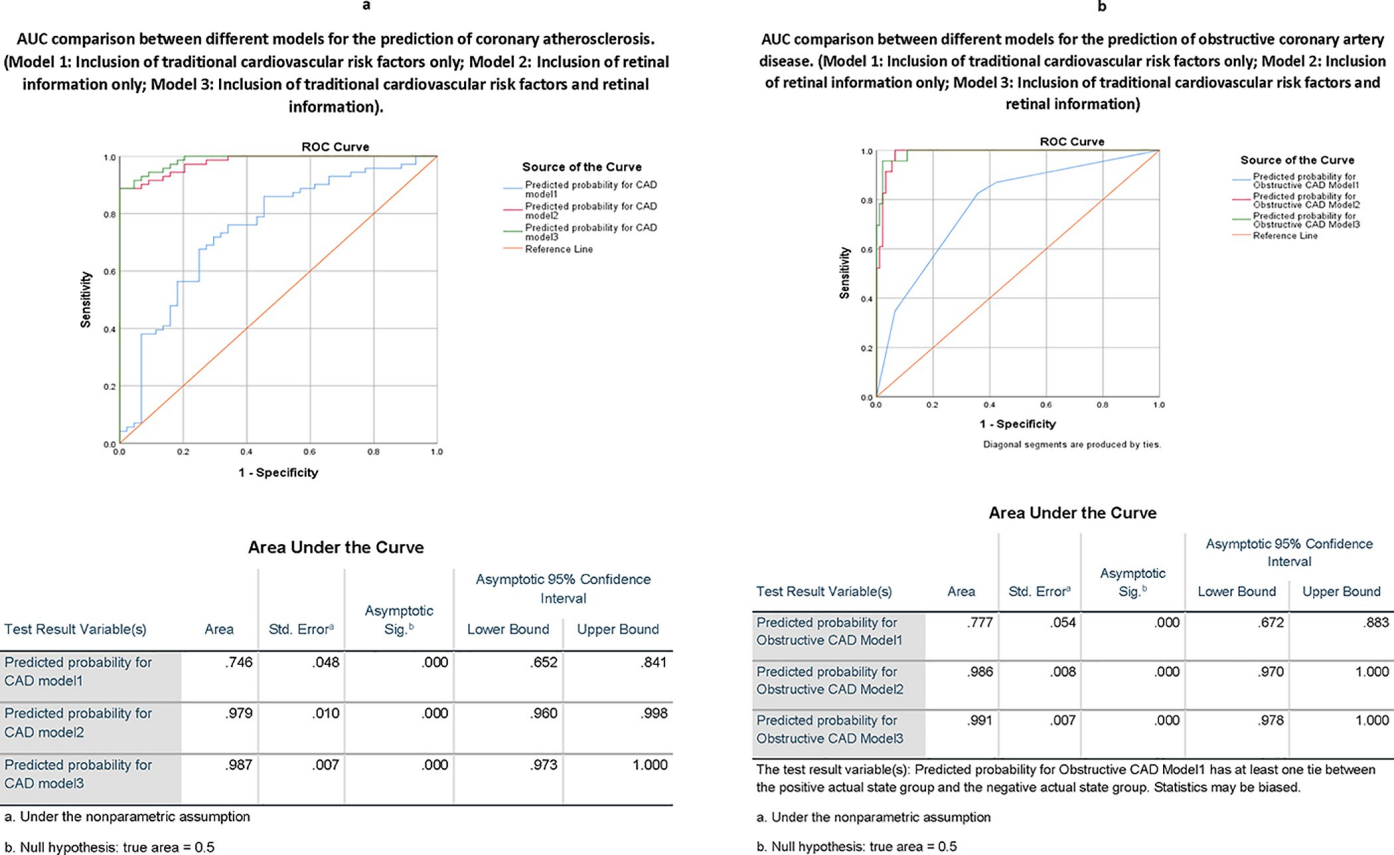
**a.** AUC comparison between different models for the prediction of coronary atherosclerosis. (Model 1: Inclusion of traditional cardiovascular risk factors only; Model 2: Inclusion of retinal information only; Model 3: Inclusion of traditional cardiovascular risk factors and retinal information). **b.** AUC comparison between different models for the prediction of obstructive coronary artery disease. (Model 1: Inclusion of traditional cardiovascular risk factors only; Model 2: Inclusion of retinal information only; Model 3: Inclusion of traditional cardiovascular risk factors and retinal information).

The retinal characteristics associated with coronary atherosclerosis, obstructive CAD, and significant CAC are shown in **S2 and S3 Tables**. Several retinal vascular characteristics were associated with coronary atherosclerosis. They include narrower arterioles, wider venules, and a lower degree of arteriolar branching,

The associations between traditional cardiovascular risk factors and retinal characteristics with coronary atherosclerosis are shown in **Table 3**. We further improve the classification using a machine-learning approach. The model, including traditional cardiovascular risk factors and retinal characteristics, had 93.0% sensitivity and 93.2% specificity (area under the ROC curve (AUC) was 0.987, 95% CI 0.973–1.00). The performance is similar to the model with retinal characteristics alone, with 91.5% sensitivity and 88.6% specificity (AUC 0.979, 95% CI 0.960–0.998). However, both models with retinal characteristics were better than the model with traditional cardiovascular risk factors alone, with 81.7% sensitivity and 54.5% specificity (AUC 0.746, 95% CI 0.652–0.841) in assessing the risk of coronary atherosclerosis (**Fig 2A**).

**Table 3:** Stepwise logistic regression analysis showing factors associated with coronary atherosclerosis in different models.

	Odds Ratio (OR)	95% CI for OR		P-value
		Lower	Upper	
**Model 1: Inclusion of traditional cardiovascular risk factors only**				
Dyslipidemia	3.30	1.41	7.77	0.006
CD4:CD8 ratio	0.21	0.07	0.68	0.009
Hypertension	2.56	1.09	6.04	0.031
AUC: 0.7465 (95%CI: 0.6514–0.8416)				
**Model 2: Inclusion of retinal characteristics only**				
Left MBCA	0.69	0.46	1.04	0.079
Right CRVE	2.16	1.10	4.24	0.025
Right adjusted CRAE	0.32	0.15	0.65	0.002
Right MV asymmetry	0.58	0.36	0.92	0.020
AUC: 0.7298 (95%CI: 0.6356–0.8241)				
**Model 3: Inclusion of traditional cardiovascular risk factors and retinal characteristics retinal characteristics**				
Dyslipidemia	3.27	1.31	8.17	0.011
CD4:CD8 ratio	0.23	0.07	0.79	0.019
Hypertension	2.23	0.88	5.62	0.089
Left MBCA	0.66	0.42	1.04	0.075
Right CRVE	2.04	0.98	4.24	0.057
Right adjusted CRAE	0.34	0.16	0.74	0.007
Right MV asymmetry	0.61	0.38	1.00	0.049
AUC: 0.8073 (95%CI: 0.7216–0.893)				

Abbreviations: CRAE, central retinal arteriolar equivalent; CRVE, central retinal venular equivalent; MBCA, bifurcation coefficient of artery; MV asymmetry, venous asymmetry index.

The models showing the associations between traditional cardiovascular risk factors plus retinal characteristics for obstructive CAD are shown in **Table 4**. For assessing the risk of obstructive CAD, the model including retinal variables combined with traditional risk factors had a sensitivity and specificity of 95.7% and 97.8%, respectively (AUC 0.991, 95% CI 0.978–1.000). The model with retinal characteristics alone had sensitivity and specificity of 87.0% and 96.7%, respectively (AUC 0.986, 95% CI 0.970–1.000). Both models, including retinal characteristics, performed significantly better than the model with traditional risk factors alone and had sensitivity and specificity of 34.3% and 93.5%, respectively (AUC 0.777, 95% CI 0.672–0.883) (**Fig 2B**).

**Table 4:** Stepwise logistic regression analysis showing factors associated with obstructive coronary artery disease in different models.

	OR	95% CI for OR		P-value
		Lower	Upper	
**Model 1: Inclusion of traditional cardiovascular risk factors only**				
Age ≥55 years	7.39	2.28	24.01	0.001
Dyspnea	3.20	1.04	9.79	0.042
AUC: 0.7774 (95%CI: 0.6777–0.8771)				
**Model 2: Inclusion of retinal characteristics only**				
Left MBCV	2.60	1.30	5.20	0.007
Left AVR	1.11	1.03	1.20	0.006
Right MBCA	0.14	0.05	0.42	< .001
Right AF	2.97	1.03	8.54	0.044
Right Tortuosity	0.39	0.17	0.92	0.030
Right A occlusion	2.91	1.17	7.27	0.022
AUC: 0.9022 (95%CI: 0.8098–0.9946)				
**Model 3: Inclusion of traditional cardiovascular risk factors and retinal characteristics retinal characteristics**				
Dyspnoea	5.82	1.03	32.95	0.047
Left MBCV	2.36	1.14	4.87	0.020
Left AVR	1.11	1.03	1.21	0.011
Right MBCA	0.16	0.05	0.50	0.001
Right AF	3.27	1.05	10.17	0.041
Right Tortuosity	0.34	0.13	0.86	0.023
Right A occlusion	2.96	1.17	7.49	0.022
AUC: 0.9168 (95%CI: 0.8420–0.9917)				

Abbreviations: MBCA, bifurcation coefficient of artery; MBCV, bifurcation coefficient of venule; AF, atrial fibrillation; Tortuosity, Tortuosity; A occlusion, Arteriole occlusion; AVR, Arteriole-venule ratio.

**Fig 3:**
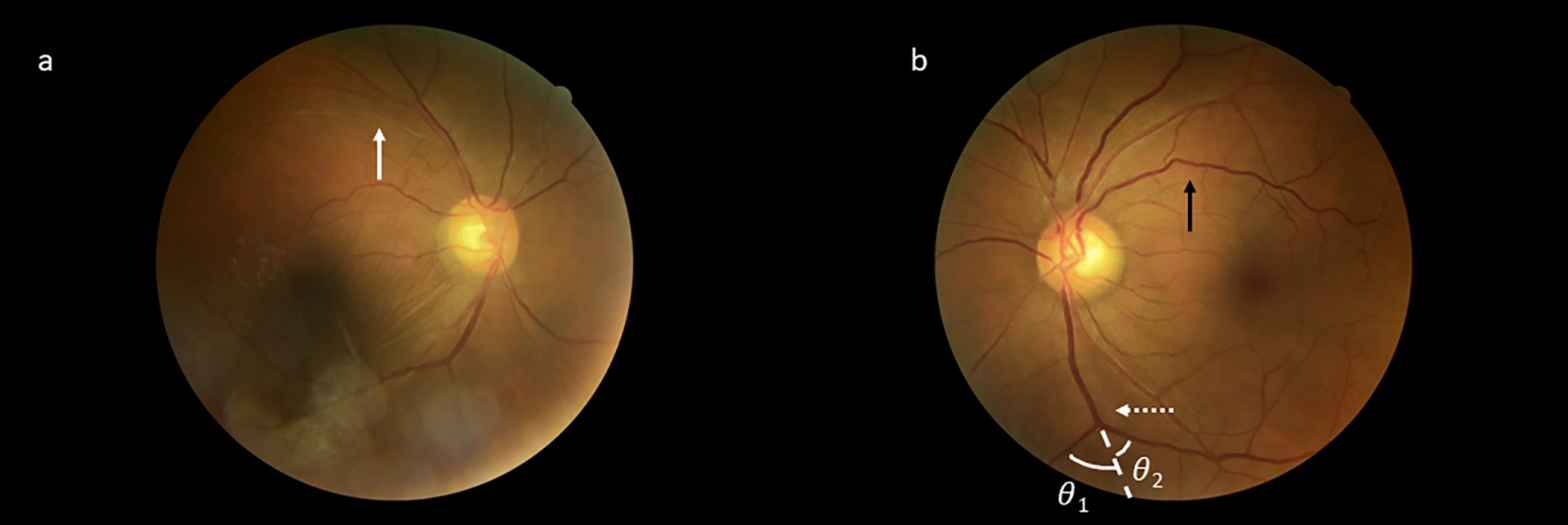
a & b show the retinal images of two participants with coronary artery disease. White arrow indicates arteriole occlusion. Black arrow indicates a venous vessel with significant vascular tortuosity. The dashed white arrow indicates a venous bifurcation; *θ*_1_ and *θ*_2_ specify the bifurcation coefficients of the two branching venules (MBCV).

Likewise, models including retinal characteristics alone or those combined with traditional cardiovascular risk factors performed significantly better than those with traditional risk factors alone in assessing the risk of significant CAC (**S4 Table** and **S1 Fig)**. An example of retinal vascular characteristics and their related measurements is shown in **Fig 3**.

All conventional cardiovascular disease risk prediction functions had limited performance in assessing the risk of coronary atherosclerosis and obstructive CAD. Among those participants with coronary atherosclerosis, 45.1%, 57.7%, 52.1% and 42.3% were categorized as having low risk using Framingham risk score, QRISK3, Pooled Cohort ASCVD risk equations, and 10-year DAD cohort risk prediction, respectively. The corresponding figures for obstructive CAD among these groups were 30.4%, 52.2%, 34.8% and 34.8%, respectively. Moreover, ROC curve analyses showed that all of the prediction functions had AUC <0.7, with QRISK3 having the highest AUC for assessing both coronary atherosclerosis (AUC 0.667, 95% CI 0.566–0.767) and obstructive CAD (AUC 0.663, 95% CI 0.545–0.780) (**S5 Table**). For fairness in comparison, we used only the logistic regression model as a method for the comparison without using the machine-learning classification method. The models adopting the retinal characteristics alone (AUC 0.902, 95% CI 0.810–0.995) or in combination with the QRISK3 score (AUC 0.912, 95% CI 0.829–0.994) had a significantly better performance than QRISK3 score alone in assessing the risk of obstructive CAD (**S6 and S7 Tables** and **S2 and S3 Figs**).

## Discussion

In this study, we evaluated machine-learning-based retinal imaging analysis to assess the risk of CAD in PLWH. In this cohort of Asian PLWH at risk of developing cardiovascular diseases, retinal characteristics, either alone or combined with traditional risk factors, enhanced the performance in assessing the risk of coronary atherosclerosis and obstructive CAD. In contrast, the performance of traditional cardiovascular risk prediction functions has a lot of room to improve.

This study showed that 62% of at-risk PLWH had coronary atherosclerosis, and 20% had obstructive CAD. The most extensive cohort study involving PLWH in Asia had recently demonstrated that traditional cardiovascular risk factors, including older age, hypertension, dyslipidemia, and high body mass index, were the major contributing factors for the development of cardiovascular disease diseases among PLWH in the region [32]. Our study further confirmed the high prevalence of CAD and its contributing risk factors among PLWH, highlighting the importance of prevention and treatment of these risk factors among PLWH. Moreover, an accurate tool to predict cardiovascular risk in these at-risk individuals is highly recommended.

We demonstrated that all of the evaluated cardiovascular risk prediction functions had limited accuracy in assessing the risks of coronary atherosclerosis and obstructive CAD in Asian PLWH. Evidence from current literature suggests that currently available cardiovascular risk prediction functions have suboptimal performance among PLWH. For example, Framingham risk scores tended to underestimate the prevalence of CAD and other atherosclerotic cardiovascular diseases among PLWH in the United States across all risk groups [33] while overestimating cardiovascular risk in European [34] and Asian [35] populations of PLWH. The available HIV-specific DAD cohort risk-prediction model was developed in a primarily European cohort [6]. It predicted a lower proportion having a high risk of cardiovascular disease among Asian PLWH than other prediction functions [35–37]. However, to the best of our knowledge, it had not yet been validated in any Asian populations of PLWH. Better tools to accurately assess the risk of CAD and other cardiovascular diseases among Asian PLWH are preferred.

Currently available studies performed in general HIV-uninfected populations have demonstrated the association of retinal vascular characteristics with multiple cardiovascular risk factors, including body mass index, smoking, hypertension, diabetes, and dyslipidemia [12, 13, 38, 39]. To supplement, these studies included multi-ethnic populations, including China [12], Britain [13], and the Middle East [38, 39]. In particular, smaller arteriolar widths, larger venular widths, and increased arteriolar and venular tortuosity were associated with cardiovascular risk factors [13]. Furthermore, using deep learning on retinal images, it was able to predict the presence of CAC and stratify cardiovascular disease risk in multi-ethnic populations [40]. In addition, the retinal vascular density and vessel branching complexity were able to predict higher mortality incidents in populations with high rates of hypertension and diabetes [15].

Regarding the prediction models of CAD, longitudinal studies showed that arteriolar narrowing, venular widening and fractal dimension accurately predicted incident CAD and CAD mortality in population-based studies [14, 41]. Patients hospitalized with acute coronary syndrome had lower retinal inner vessel length density and perfusion density than controls with lower cardiovascular risk [42]. In another study from China, patients with stable CAD had lower vessel density in superficial capillary plexus and deep capillary plexus; vessel density was also associated with CAD severity [43]. To further understand the pathogenesis of retinal vessel disease, recent genome-wide association analyses identified loci associated with retinal microvascular architecture, including genes associated with inflammatory, chemokine and angiogenesis pathways [15]. Such associations suggested that the retina may provide a window for identifying pathogenic processes underlying cardiovascular diseases in both HIV-uninfected populations and PLWH.

Our study further showed that a machine-learning-based retinal image analysis could improve the accuracy of assessing CAD risk among PLWH. In addition, it enhanced the performance of several widely adopted cardiovascular risk prediction functions in PLWH. This observation further supports that the retinal vessels provide an in-vivo examination of vascular sequelae secondary to CAD risk factors, including hypertension and diabetes. In studies performed in HIV-uninfected populations, retinal image analysis, when coupled with risk prediction functions, had better performance than risk prediction models alone in cardiovascular risk stratification [40] and prediction of cardiovascular mortality [44]. Retinal image analysis can potentially enhance the current risk prediction functions in cardiovascular disease risk stratification among PLWH.

The performance of retinal image analysis was best in assessing the risk of obstructive CAD in our cohort. While retinal characteristics can act as systemic biomarkers [8], the results from our study supported that retinal characteristics would be most helpful in identifying at-risk PLWH with obstructive CAD. This group of patients may benefit from more stringent cardiovascular risk factor control and coronary interventions.

Our study has several limitations. First, we had a relatively small sample size and involved only PLWH with cardiovascular risk factors from Asia. The cross-sectional study design precluded the evaluation of prediction of incident cardiovascular events by retinal image analysis. Future studies should evaluate the performance of retinal image analysis in predicting CAD in a more diverse population of PLWH with different ethnicities and levels of cardiovascular risk. Previous studies have shown differences in vascular calibre and fractal dimension among Asian ethnic groups [45]. Finally, this study has not involved the use of optical coherence tomography angiography (OCTA), which can provide more detailed retinal and choroidal characteristics analyses, which have also been shown to be associated with CAD [46].

In conclusion, we have demonstrated that machine-learning-based retinal image analysis accurately assesses the risk of coronary atherosclerosis and obstructive CAD among PLWH with risk factors for cardiovascular diseases. This tool should be further validated in more diverse populations of PLWH for the adoption in clinical practice for CAD risk stratification.

## Supporting information

S1 Table Demographic and clinical characteristics in participants with and without significant coronary artery calcium. (TIF)

S2 Table Retinal characteristics in participants with and without coronary atherosclerosis or obstructive CAD. (TIF)

S3 Table Retinal characteristics in participants with and without significant coronary artery calcium. (TIF)

S4 Table Stepwise logistic regression analysis shows factors associated with significant coronary artery calcium in different models. (TIFF)

S5 Table Area under curve (AUC) of cardiovascular risk prediction functions for coronary atherosclerosis and obstructive coronary artery disease. (TIFF)

S6 Table Stepwise logistic regression analysis showing risk prediction function and retinal characteristics associated with coronary atherosclerosis in different models. (TIFF)

S7 Table Stepwise logistic regression analysis showing risk prediction function and retinal characteristics associated with obstructive coronary artery disease in different models. (TIFF)

S1 Fig Machine-learning analysis for AUC comparison between different models in the prediction of significant CAC (Model 1: Inclusion of traditional cardiovascular risk factors only; Model 2: Inclusion of retinal information only; Model 3: Inclusion of traditional cardiovascular risk factors and retinal information). (TIFF)

S2 Fig Machine-learning analysis for AUC comparison between different models including risk prediction function and retinal information in predicting coronary atherosclerosis. (Model 1: Inclusion of cardiovascular risk prediction function with the highest AUC only; Model 2: Inclusion of retinal information only; Model 3: Inclusion of cardiovascular risk prediction score and retinal characteristics).
(TIFF)

S3 Fig Machine-learning analysis for AUC comparison between different models including risk prediction score and retinal information in predicting obstructive coronary artery disease. (Model 1: Inclusion of cardiovascular risk prediction score with the highest AUC only; Model 2: Inclusion of retinal information only; Model 3: Inclusion of cardiovascular risk prediction score and retinal characteristics).
(TIFF)
