# Supplementary figures and images for: An efficient approach to estimate the risk of coronary artery disease for people living with HIV using machine-learning-based retinal image analysis
Source: PLoS One. 2023 Feb 24;18(2):e0281701. doi: 10.1371/journal.pone.0281701 (PMC9955663; doi:10.1371/journal.pone.0281701)

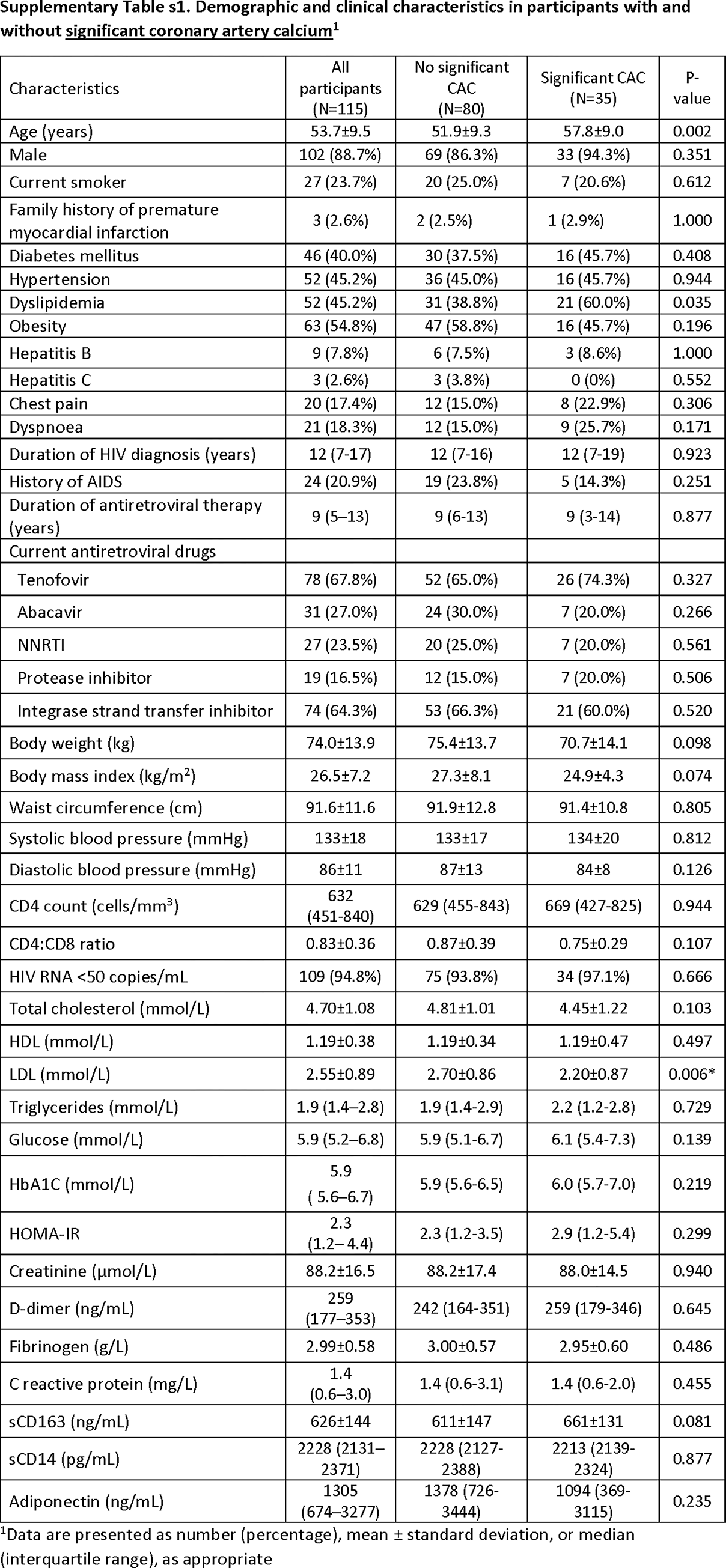

Supplement: S1 Table — (TIF) [file pone.0281701.s001.tif]

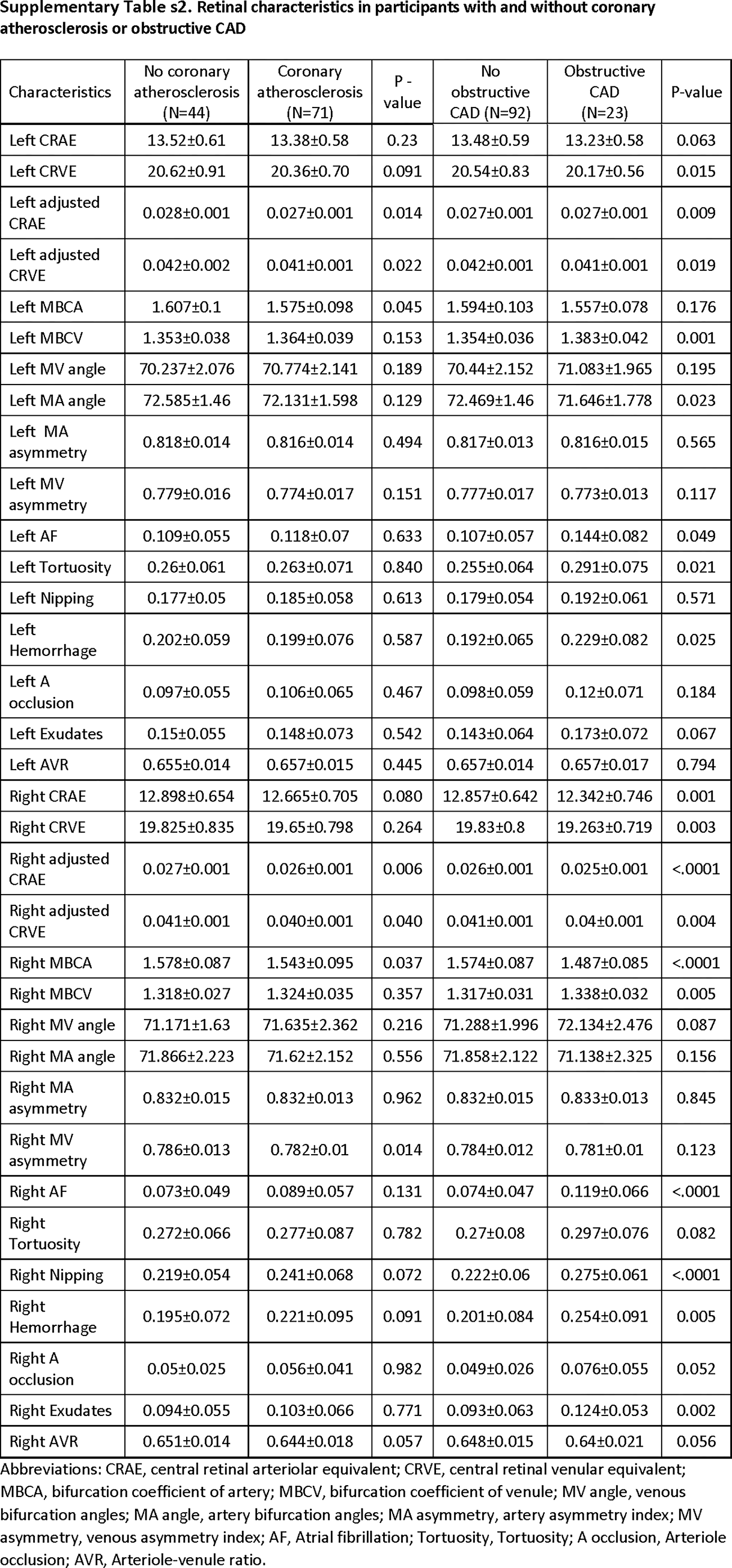

Supplement: S2 Table — (TIF) [file pone.0281701.s002.tif]

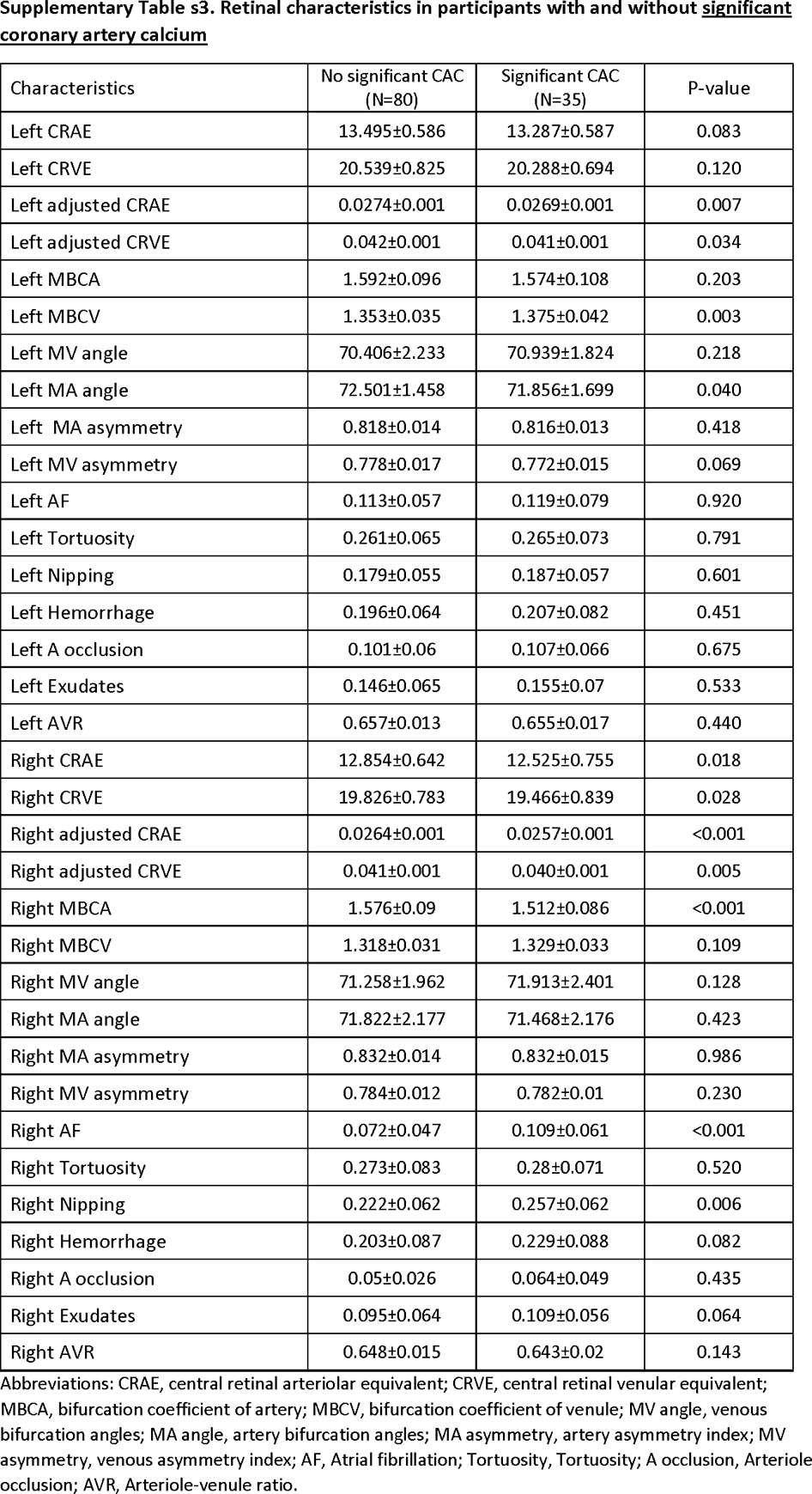

Supplement: S3 Table — (TIF) [file pone.0281701.s003.tif]

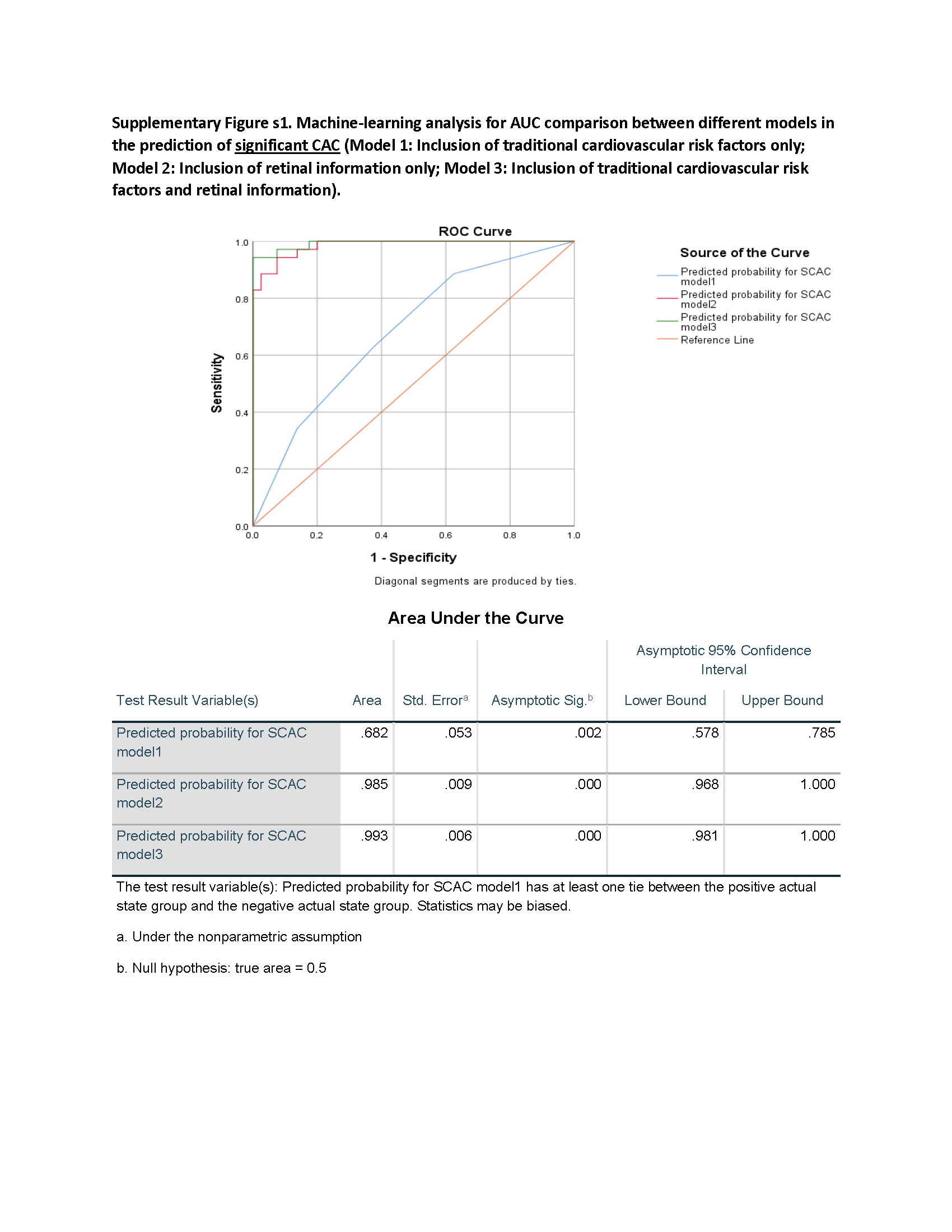

Supplement: S1 Fig — (TIFF) [file pone.0281701.s008.tiff]

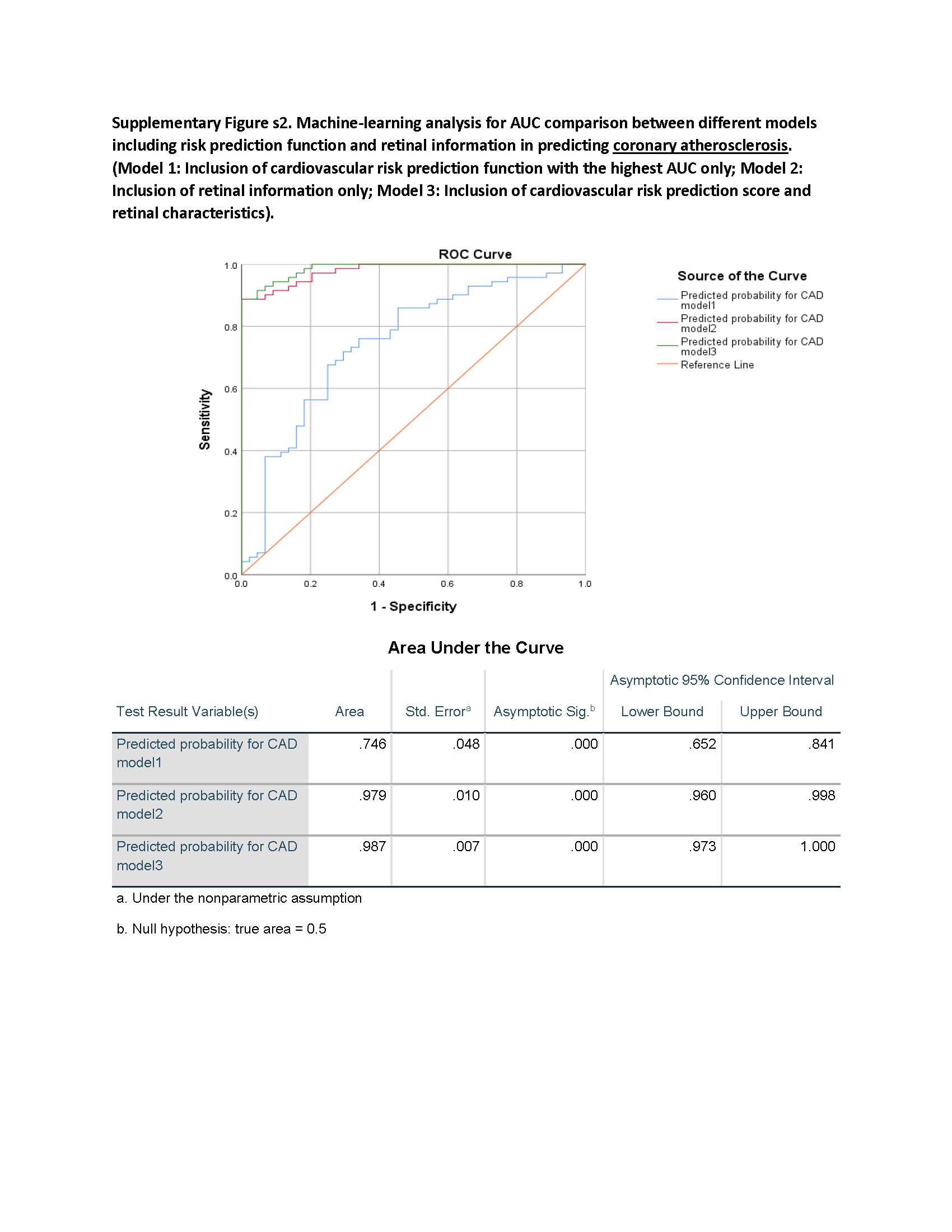

Supplement: S2 Fig — (Model 1: Inclusion of cardiovascular risk prediction function with the highest AUC only; Model 2: Inclusion of retinal information only; Model 3: Inclusion of cardiovascular risk prediction score and retinal characteristics). (TIFF) [file pone.0281701.s009.tiff]

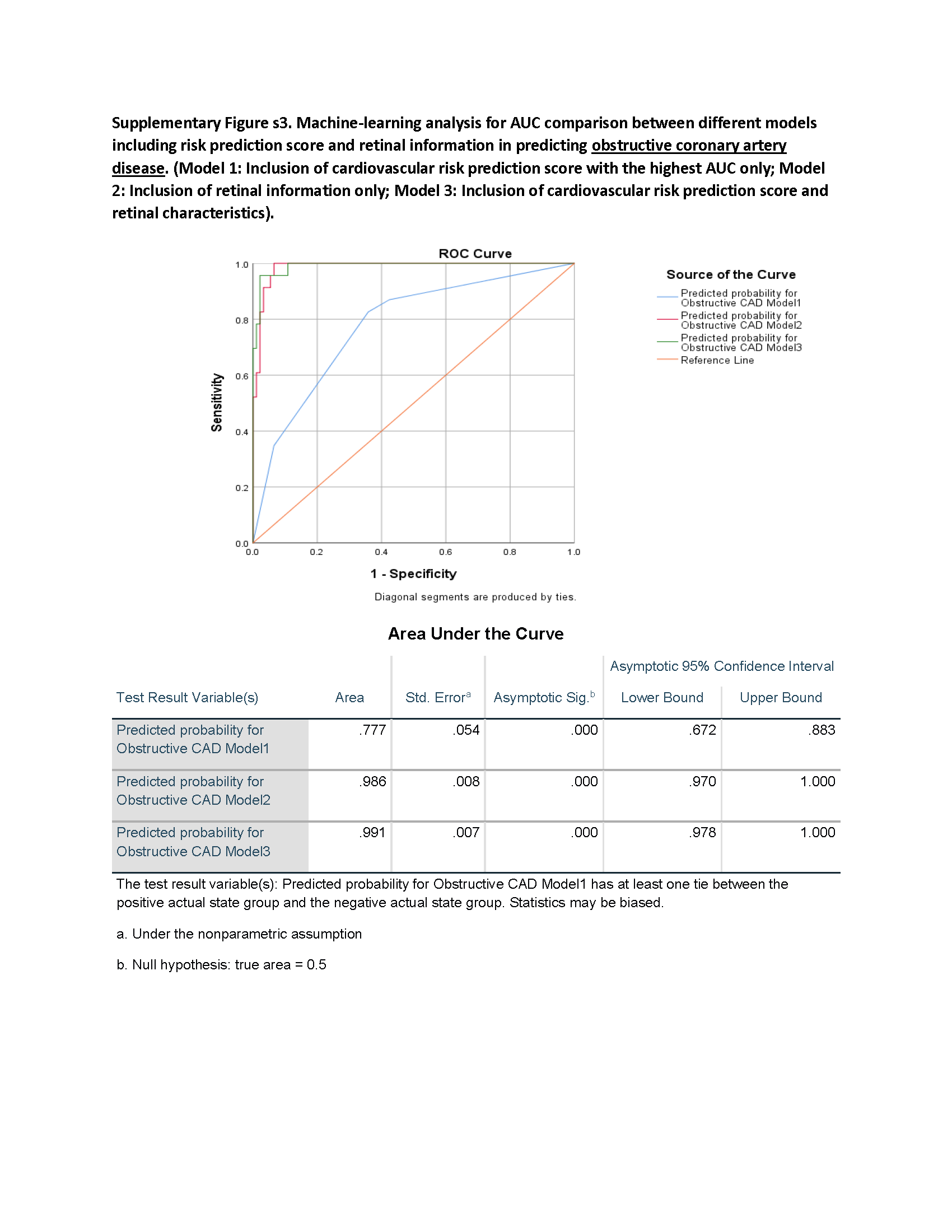

Supplement: S3 Fig — (Model 1: Inclusion of cardiovascular risk prediction score with the highest AUC only; Model 2: Inclusion of retinal information only; Model 3: Inclusion of cardiovascular risk prediction score and retinal characteristics). (TIFF) [file pone.0281701.s010.tiff]
